# Reemergence of diphtheria in France: Description of 3 cutaneous cases

**DOI:** 10.1016/j.jdcr.2024.07.040

**Published:** 2024-08-30

**Authors:** Clémence Boucher, Ali Dadban, Eulalie Laude Pagniez, Anna Potereau, Florian Lombart, Jean-Philippe Arnault, Guillaume Chaby

**Affiliations:** Department of Dermatology, Amiens University Hospital, Amiens, France

**Keywords:** *Corynebacterium diphtheriae*, diphtheria, emergence, refugee

## Introduction

Diphtheria is a toxin-mediated infection mainly caused by *Corynebacterium diphtheriae* and *Corynebacterium ulcerans*, which are members of the *Corynebacterium* species that are gram-positive bacillus. Transmission is human-to-human, via droplets from the upper respiratory tract or through skin contact.^1^ Historically, the disease presents with pharyngitis with pseudomembrane on the tonsils and throats.^2^ The cutaneous form, however, represents an important mode of dissemination, because it is most often unrecognized and diagnosed late.^2^

In France, since 1990, a widespread vaccination against diphtheria has led to the disappearance of this infection.^3^ However, the disease remains a major public health problem in some regions of the world where vaccination coverage is insufficient. They are the main sources of imported cases in Western countries.^3^

After the COVID pandemic, immunization coverage dropped to its lowest level since 2008.^4^ Over the past decade, the European Centre for Disease Control and Prevention reports a significant increase in the number of cases in several countries in Europe.^3^ The presentation of the cases was mainly in cutaneous forms, most commonly in young male adults.^4^

In this article, we report 3 cases of cutaneous diphtheria, mistaken for cutaneous staphylococcal infection led to delaying the diagnosis.

## Case 1

**Fig 1:**
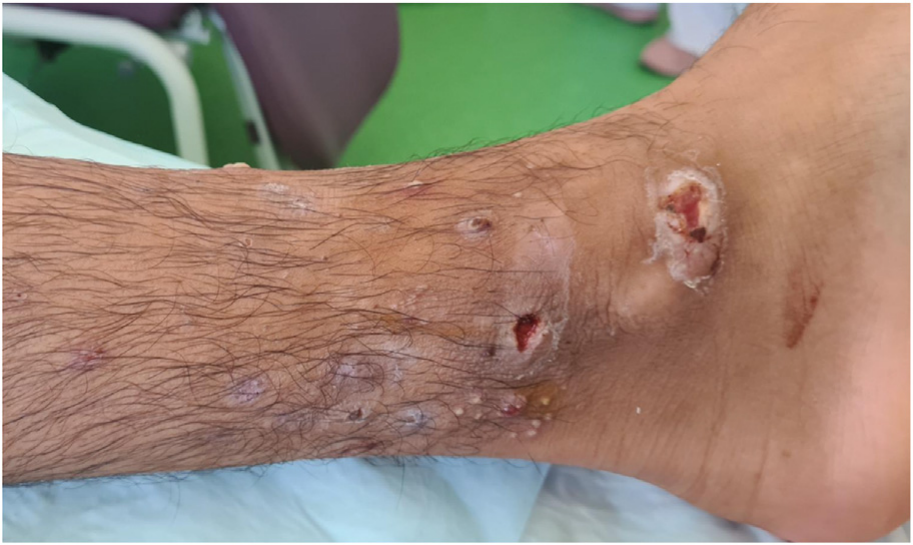
Pustules and ulcerations of the distal extremity of the left leg (patient 1).

**Fig 2:**
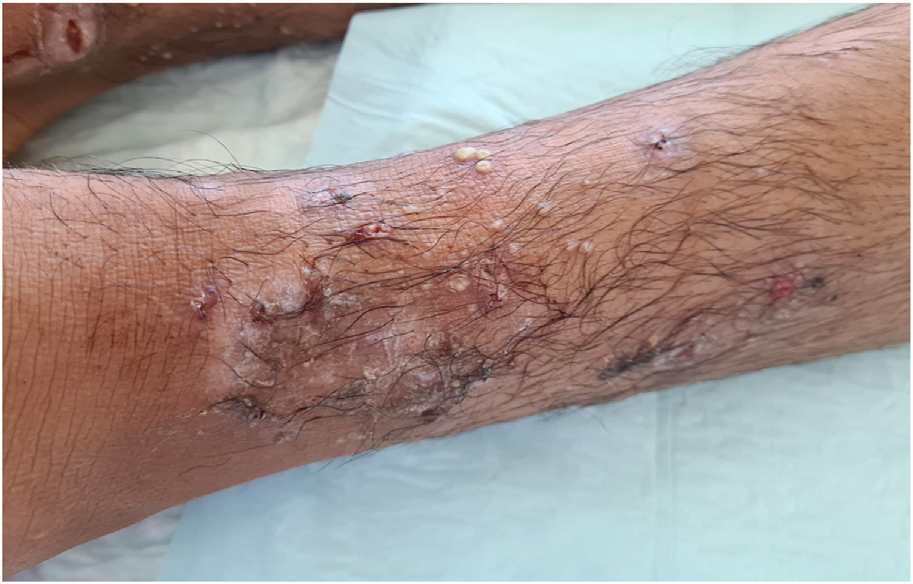
Pustules and scabs of the distal extremity of the right leg (patient 1).

The first patient was a 15-year-old refugee boy of Afghan origin with unremarkable past medical history. During his journey to France, pustular lesions developed, sometimes umbilicated, which progressed to punched-out, crusted ulcerations. They were located at the distal ends of the lower limbs and had been present for 1 month, with low-grade fever at 38 °C (Figs 1 and 2). The rest of the clinical examination did not reveal pharyngitis or lymphadenopathy. Antibiotic therapy with amoxicillin/clavulanic acid for 8 days had led to slight improvement of these lesions, which became worse after stopping the antibiotics. Blood counts, renal function and liver function tests were unremarkable. Bacteriological and mycological skin samples were initially negative. The patient was hospitalized and a treatment by oral clindamycin 600 mg 3 times a day was started. However, the patient had eloped during the fourth day of hospitalization and the follow-up was lost.

## Case 2

**Fig 3:**
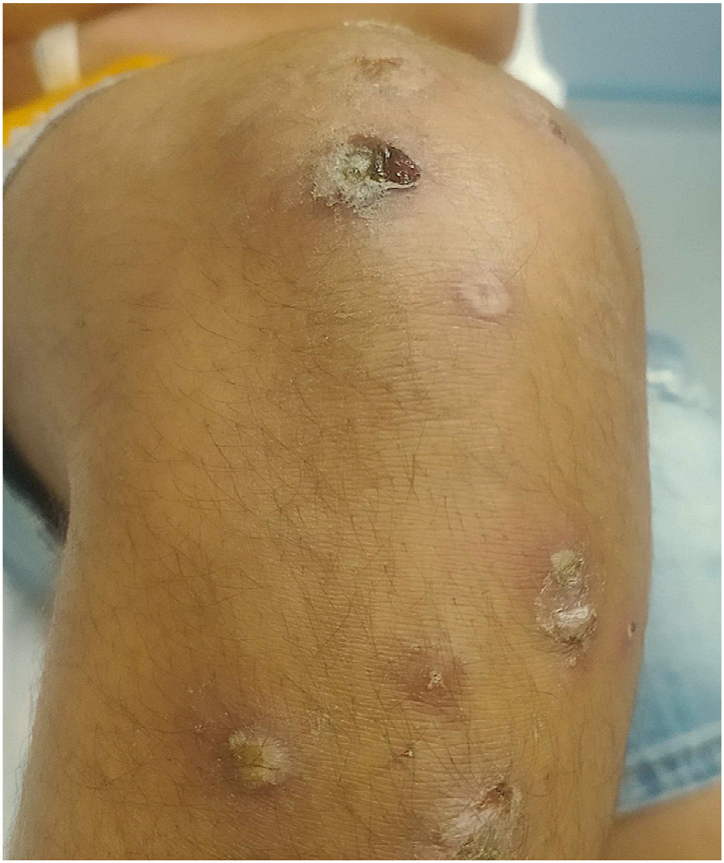
Pretibial crusts and ulcerations (patient 2).

**Fig 4:**
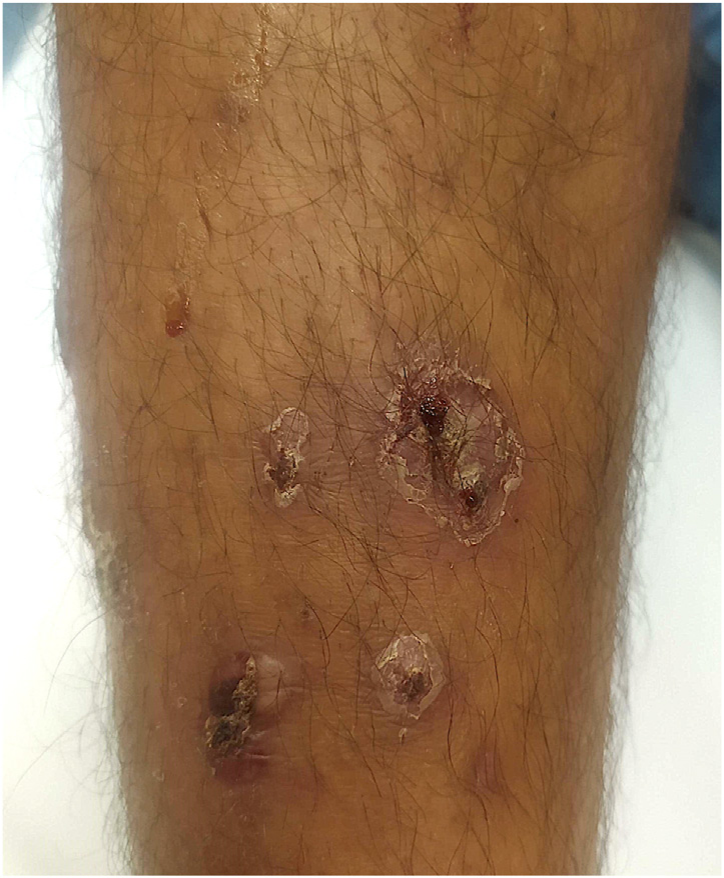
Pustules and scabs of the right knee (patient 2).

The second patient, aged 17 year old boy also of Afghan origin, consulted for crusted, small ulcers on both legs with some pustules (Figs 3 and 4). His general condition was good, without fever or pharyngitis. With help from a translator, the patient confirmed that he was a refugee and was in contact with the first patient during his journey to France (case 1) a few days earlier. The bacteriological skin sample came back positive for *C diphtheriae*. Antibiotic therapy with erythromycin 1 g 3 times a day for 14 days combined with remote vaccination allowed complete healing of the lesions. A new culture on specific medium (blood agar) of skin samples from the first patients was requested and found evidence of *C diphtheriae.* First patient eloped and we were unable to contact him to complete his treatment.

## Case 3

**Fig 5:**
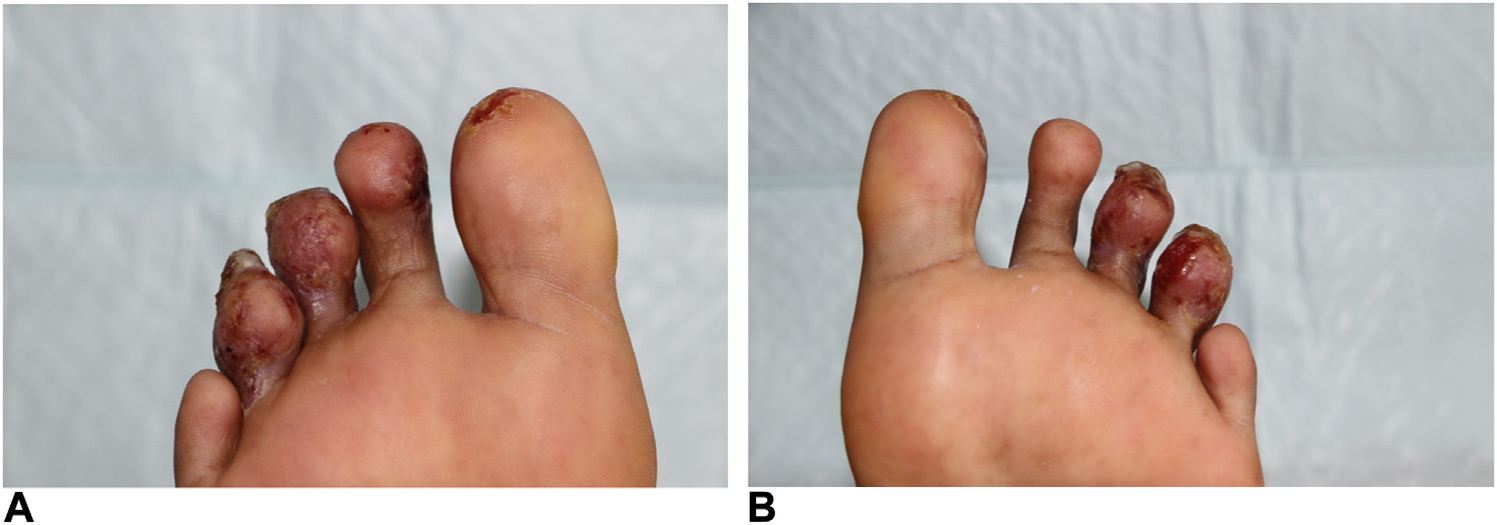
Ulceration of the pads of the toes of both feet (patient 3).

The third patient was a 36-year-old woman of Malagasy origin and living in France for many years with painful ulcerative lesions of the toe pulp bilaterally evolving since 7 months (Fig 5). The initial appearance was pustular with a secondary progression to ulceration. The rest of the clinical examination was unremarkable, with no signs of pharyngitis in particular. Various antibiotic therapies had been introduced by the treating physician, including amoxicillin, with no improvement.

Further history taking showed that these skin lesions had appeared during a trip to Madagascar. Bacteriological samples taken from the skin of the toes revealed *Staphylococcus aureus* (sensitive to amoxicillin), *Streptococcus pyogenes* (sensitive to amoxicillin), and nontoxin secreting *C diphtheriae* (resistant to amoxicillin). Nasopharyngeal swabs revealed *C diphtheriae* (resistant to amoxicillin). Antibiotic therapy with clindamycin 600 mg × 3 resulted in healing of the lesions. Vaccination schedule was later updated. The patient’s entourage were offered an update of the diphtheria, tetanus and pertussis vaccination.

## Discussion

Cutaneous diphtheria is an ancient disease that is re-emerging since recent years. It may be due to toxigenic or nontoxigenic corynebacteria. The 2 most frequently causative germs are *C diphtheriae* and *C ulcerans*. France, such as other countries in Europe, is currently experiencing an increase in the number of cases of diphtheria, especially the cutaneous form. From January 2022 to February 2, 2023, 273 cases of diphtheria among migrants were reported by 8 European Union countries.^5^ Among these cases, more than two-thirds presented only with the cutaneous form of the disease. In France, whereas only 10 cases of toxigenic diphtheria were reported between 1989 and 2015,^6^ Public Health France reported 60 cases of diphtheria in 2022.^3^ The main hypotheses that can explain this phenomenon of reemergence are traveling, a significant decline in vaccination coverage around the world, which contrasts with the significant increase in the number of refugees subjected to poverty and promiscuity, particularly in migrant reception centers.^7^^,^^8^ Most infections were linked to *C diphtheriae*, of which 35 cases were in France. Disease manifestations were cutaneous in 27 patients, respiratory in 5 and asymptomatic in 3. They were predominantly male (*N* = 32), aged 11 to 59 years with an average age of 23 years. Symptoms appeared <15 days after their arrival in France. Only 3 patients were up to date with their diphtheria vaccination. For the other 25 patients, vaccination status was unknown. Most cases were diagnosed in migrants from Afghanistan (*n* = 28) or travelers (*n* = 6).

*C diphtheriae* is most often transmitted through secretions from the respiratory tract. In rare cases, transmission can occur by direct contact with a skin lesion or with soiled items contaminated with discharge from these lesions.^9^

The incubation period for cutaneous diphtheria ranges from 2 to 5 days (can range from 1 to 10 days).^9^

The diagnosis of cutaneous diphtheria can be challenging for dermatologists because skin manifestations are vague and nonspecific.

Most often after injuries to the limbs, they initially manifest as pustules, which then develop into very limited ulcers covered by a pseudomembrane, with few general symptoms.^10^ Skin lesions are usually mistaken for impetigo, but when pustules and crusts on the leg are seen, spaced apart and without bullae, the diagnosis of cutaneous diphtheria should be considered.

Concomitant nasopharyngeal infection occurs in 20% to 40% by direct or indirect contamination from pre-existing chronic skin lesions.^11^

Diphtheria’s severity depends on myocardial or neurologic involvement of toxin-secreting strains of *Corynebacterium* producing diphtheria toxin.^7^ Toxin effects rarely occur in cutaneous diphtheria, with an incidence of 1% to 2%, because of the slower release of the toxin across the skin barrier.^12^

The diagnosis is confirmed by culture of a skin smear on a standard culture medium but is better detected on a special culture media (blood agar, Loeffler, or Tindale).^11^ It is important to guide the bacteriologist to the correct diagnosis. Association with other bacteria, such as group A *streptococci* and/or *staphylococci aureus*, as in our patients, may mask the role of *C diphtheriae*.^13^ Samples are often returned as contaminated, as in the case of our first patient, where a new analysis was necessary.

Treatment of cutaneous diphtheria does not require diphtheria antitoxin. It is based on 2 principles: antibiotic therapy (amoxicillin or erythromycin) for 10 days and vaccine update at distance from the infection in unvaccinated subjects.^11^ Preventive antibiotics for household members and close contacts should be considered. No immunity is acquired through this infection.^11^ Resistance to amoxicillin can be found, as in our third patient, explaining the failure of treatment.^14^

It is important to note that the diphtheria vaccine is diphtheria toxoid, derived from the toxin *C diphtheriae*, which is responsible for the clinical manifestations of the disease and especially its complications.

As a result, the vaccine protects vaccinated people against severe manifestations of the toxin (cardiac, neurological, and nephrological), but does not prevent asymptomatic infections and transmission of the bacteria nor the cutaneous form of this disease.

As a result, vaccinated individuals can become infected with the bacteria without developing classic symptoms and pass it on to those around them.^8^

## Conclusion

Diphtheria is a re-emerging disease, with a significant increase in the number of cutaneous cases. The diagnosis should be suggested in the presence of ≥1 atypical ulcerations of the limbs or distal extremities upon return from a trip to an endemic area. The diagnosis must be made in the presence of an impetigo that does not respond to treatment with conventional antibiotics. Vaccination protects against severe toxigenic forms, but not against cutaneous forms. Therefore, cutaneous diphtheria may be considered even in vaccinated people.

## Conflicts of interest

None disclosed.
